# Using aggregated single patient (N-of-1) trials to determine the effectiveness of psychostimulants to reduce fatigue in advanced cancer patients: a rationale and protocol

**DOI:** 10.1186/1472-684X-12-17

**Published:** 2013-04-23

**Authors:** Hugh EJ Senior, Geoffrey K Mitchell, Jane Nikles, Sue-Ann Carmont, Philip J Schluter, David C Currow, Rohan Vora, Michael J Yelland, Meera Agar, Phillip D Good, Janet R Hardy

**Affiliations:** 1 Discipline of General Practice, The University of Queensland, Brisbane, Queensland, Australia; 2 School of Health Sciences, University of Canterbury, Christchurch, New Zealand; 3 School of Nursing and Midwifery, The University of Queensland, Brisbane, Queensland, Australia; 4 Discipline of Palliative and Supportive Services, Flinders University, Adelaide, South Australia, Australia; 5 Department of Palliative Care, Gold Coast Hospital, Gold Coast, Queensland, Australia; 6 School of Medicine, Griffith University, Gold Coast, Queensland, Australia; 7 Department of Palliative Care, St. Vincent‘s Hospital, Brisbane, Queensland, Australia; 8 Mater Health Services, Department of Palliative Care, Brisbane, Queensland, Australia

**Keywords:** Fatigue, Cancer, Methylphenidate hydrochloride, N-of-1 trial, Single patient trial, Palliative

## Abstract

**Background:**

It is estimated that 29% of deaths in Australia are caused by malignant disease each year and can be expected to increase with population ageing. In advanced cancer, the prevalence of fatigue is high at 70–90%, and can be related to the disease and/or the treatment. The negative impact of fatigue on function (physical, mental, social and spiritual) and quality of life is substantial for many palliative patients as well as their families/carers.

**Method/design:**

This paper describes the design of single patient trials (n-of-1 s or SPTs) of a psychostimulant, methylphenidate hydrochloride (MPH) (5 mg bd), compared to placebo as a treatment for fatigue, with a population estimate of the benefit by the aggregation of multiple SPTs. Forty patients who have advanced cancer will be enrolled through specialist palliative care services in Australia. Patients will complete up to 3 cycles of treatment. Each cycle is 6 days long and has 3 days treatment and 3 days placebo. The order of treatment and placebo is randomly allocated for each cycle. The primary outcome is a reduction in fatigue severity as measured by the Functional Assessment of Cancer Therapy-fatigue subscale (FACIT-F). Secondary outcomes include adverse events, quality of life, additional fatigue assessments, depression and Australian Karnovsky Performance Scale.

**Discussion:**

This study will provide high-level evidence using a novel methodological approach about the effectiveness of psychostimulants for cancer-related fatigue. If effective, the findings will guide clinical practice in reducing this prevalent condition to improve function and quality of life.

**Trial registration:**

Australian New Zealand Clinical Trials Registry ACTRN12609000794202

## Background

Cancer is the second most common cause of death in Australia, accounting for 29% of all death in 2007 [1]. Predictably, the prevalence of cancer will increase with the ageing of the population. In Australia, the number of people aged 80 years and over will increase from 3.6% in 2006 to 7.9% by 2036 [2]. Therefore, the role of palliative care in caring for these patients will become increasingly important.

Cancer-related fatigue is defined as a “distressing, persistent, subjective sense of physical, emotional and/or cognitive tiredness or exhaustion, related to cancer or cancer treatment, which is not proportional to recent activity and interferes with normal functioning” [3]. In advanced cancer, the prevalence of fatigue is high at 70–90% [4-6]. Fatigue can have a high impact on function (physical, mental, social and spiritual) and quality of life (QOL) for many palliative patients and hence their families/carers. A positive benefit for both patients and their families/carers’ QOL should occur with a reduction in fatigue [7].

Methylphenidate hydrochloride (MPH) is a central nervous system stimulant. It blocks the dopamine transporter in the pre-synaptic cell membrane, thereby raising extracellular dopamine (D2) levels [8]. Its primary indication is in the treatment of Attention Deficit Hyperactivity Disorder. The USA National Cancer Care Network Guidelines on cancer-related fatigue recommend the use of psychostimulants for fatigue after ruling out other causes of fatigue, and note that the effectiveness of psychostimulants for fatigue remains investigational [3]. When the drugs are used in carefully titrated doses, side effect profiles have generally been found to be low and reverse rapidly due to the drugs’ short half-life.

### N-of-1 trials in palliative care

N-of-1 trials are multiple-cycle, double blind, placebo-controlled crossover trials using standardized measures of effect. They are usually used for testing the effectiveness of medicines in individual patients. The randomisation order is independently generated for each patient. At the end of the trial the order is revealed, and the patient response is compared against the presence or absence of the test treatment. N-of-1 trials provide the strongest evidence possible about the efficacy of a treatment in an individual patient [9]. Aggregating the results of many n-of-1 trials allows a treatment effect to be ascertained for a patient population. For details on aggregated n-of-1 methodology refer to Zucker et al. 1997, Schluter and Ware 2005 and Nikles et al. 2011 [10-12].

## Methods/design

### Study aims

The hypothesis to be tested is whether central nervous system stimulant therapy with MPH will improve fatigue in patients with advanced life-limiting disease compared to placebo. The primary objective is to determine a population and individual patient estimate of the efficacy of MPH in alleviating fatigue with advanced cancer. Secondary aims are to assess the frequency and severity of any side effects associated with the use of MPH in this population, and to provide process evaluation on the feasibility of SPTs as a routine means of conducting clinical trials in palliative care.

### Study design

**Figure 1:**
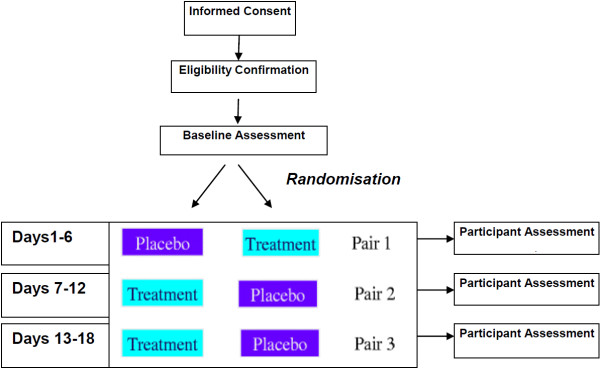
**Example of n-of-1 design schema**^**1**^**.**

This study is a series of Randomised, Double-Blind, Placebo-Controlled, Multi-Centre, Single Patient (N-of-1) Trials. In this SPT, the participant undergoes 3 pairs of treatment periods. As MPH has a short half-life (4 hours), the clinical effect is achieved quickly and a steady state is reached after 5 half-lives (i.e. 20 hours). This allows a treatment period of 3 days (and 6 days for each treatment pair), making a total of 18 days for patients to complete the full trial. No assessment of efficacy is taken on the first day of each 3-day period to allow for medication wash-out. In each cycle, drugs are randomly allocated to patients with both investigators and patient blinded (see Figure 1). At the end of the trial, the order of medications within each of the three cycles is unmasked. Repeated results from the outcome assessments in the same direction favouring the treatment can be reported in terms of a probability that the result is true.

### Study population and recruitment

Patients who are either admitted to a hospital ward or outpatients will be recruited from participating sites of the Palliative Care Clinical Studies Collaborative, namely, six specialist palliative care services in two states of Australia. Patients will be invited to participate if they are aged ≥ 18 years and have advanced cancer of any type. They must also have a Australian Karnofsky Performance Scale (AKPS) score ≥ 40, a fatigue score ≥ 4/10 by a single screening questionnaire adopted from the NCCN cancer-related fatigue guidelines, [13] a stable treatment regimen (including steroids) for at least 48 hours and likely to remain stable throughout the trial period, no planned treatment likely to influence fatigue during the trial, no change in thyroxine therapy or antidepressant therapy in the 3 weeks prior and an ability to understand all study requirements. Exclusion criteria are: unable to comprehend written English, confusion or mini-mental state examination (MMSE) < 24, unstable symptoms or disease such that the patient is unlikely to be able to complete all study requirements, history of severe ischaemic heart disease, uncontrolled cardiac arrhythmias or hypertension, electrolyte imbalances (Na, K, Mg, Ca) where attempt at correction is being considered, anaemia for which a blood transfusion is indicated, erythropoietin therapy in the previous 2 weeks.

### Primary outcome

Patients will complete a daily diary which includes the Functional Assessment of Cancer Therapy-Fatigue (FACIT-F) sub-scale [14,15]. The primary outcome is a change in the mean FACIT-F scores for patients on MPH compared to placebo. FACIT-F is a 13 question, 5 point Likert scale with a possible range of scores from 0 to 52. It is reliable, valid and sensitive to detecting the impact of fatigue in cancer patients [14,15]. A three point change in the scale is regarded as clinically significant for all cancer patients. It has also been shown to relate to self-reported capacity to perform everyday activities [16].

### Secondary outcomes

Demographics and cancer related medical history will be collected at baseline (age, gender, status of primary caregiver, tumour type, year of diagnosis, sites of metastases). Patients will complete a daily diary recording symptom scores for fatigue related symptoms using validated measures (see below) and also record any side effects. Fatigue will also be assessed by the Wu Fatigue Scale [17]. This will be compared to results from the FACIT-F subscale. The Wu Cancer Fatigue Scale (WCFS) is a valid and reliable nine question, five point scale, with a range of possible scores from five to 45. A higher score indicates greater fatigue. Depression will be assessed using the valid and reliable Edinburgh Depression Scale which consists of 10 items, rated on a four-point scale and includes items on guilt, thoughts of self-harm and hopelessness [18,19]. A higher score indicates more depression, and the tool has been validated in patients with advanced cancer [19]. Performance status will be measured at the end of the three-day cycle using the AKPS scale which has high inter-rater reliability and is sensitive to changes in function over time. A score of 0 to 100 (in increments of 10) is assigned to patients based on their ability to undertake a range of daily tasks.

### Randomisation

Each cycle will be two periods of three days each for a maximum of three cycles of 6 days. A computer generated randomisation schedule held by the pharmacy (and not accessible to investigators) will determine the order of medication (MPH or placebo) in each cycle. Randomisation is to be conducted in blocks of four to prevent unblinding.

### Safety reviews

In the patient diary and at each contact with a researcher, adverse events and medication side effects will be assessed. Patients will be contacted every 3 days, (or daily if there are any concerns) to ensure diary completion and to assess any adverse events. The severity of the side effect will be rated according to NCI Common Terminology Criteria for Adverse Events [20]. Any rating ≥ 3 of 3 or 4 in severity will activate cessation of the study intervention for that participant and an adverse event report to the Data and Safety Monitoring Board (DSMB) and the ethics committees. Common side effects are nausea, loss of appetite, anxiety, insomnia, dry mouth, tachycardia, palpitations, and changes in blood pressure (usually increases in adults). Infrequent side effects are movement disorders, tics, rash, weight loss, and growth retardation in children. Rare side effects include psychosis, neuroleptic malignant syndrome, and liver dysfunction [8]. Compliance with trial medication will be assessed by capsule count. A cycle will only be included in the analysis if all capsules for that cycle (6 days) have been taken.

### Treatment, concomitant medications and compliance

To inform this trial, a dose finding study was undertaken in 10 patients with fatigue and advanced cancer to assess the optimal dose that has an effect on fatigue [21]. Participants were commenced on a dose of 5 mg daily and the dose was increased every 3 days to a maximum of 15 mg bd. The study found that the optimal dose was 5 mg twice daily, above which there was only limited improvement in fatigue.

Patients will randomly be assigned each of the following two medications in each of the three cycles.

1. Active Medicine: MPH. Dose 5 mg encapsulated tablet bd by mouth.

2. Placebo: visually matched capsule bd by mouth.

MPH and placebo capsules will be prepared and packaged by the Pharmacy Production Unit at the lead hospital’s pharmacy in Brisbane, and couriered to the participating sites. Pre-packaged, numbered medication packs will be available at each site, and the packages specified by the randomisation will be allocated to the patient by the site hospital pharmacy upon prescription from the study investigator. Trial participants are to continue their current concomitant medicine regimen. Any changes in concomitant medications are to be documented. Participants are requested to bring all medication bottles and unused capsules back to clinic. Compliance will be assessed by capsule count. All unused study medicine will be destroyed by the hospital pharmacy on completion of study participation.

### Post-trial treatment for individual patients

Once the analysis has been completed for an individual patient at the end of the trial for a specific patient, a report detailing the single patient trial findings and recommendations is sent to the palliative care specialist for the patient. An appointment is made for the patient to discuss the results with their doctor and to make a decision regarding further treatment with MPH.

### Statistical considerations

#### Sample size

In a double-blind placebo controlled variable dose RCT trial of MPH (5–20 mg/day over 7 days) for advanced cancer-related fatigue, the mean baseline FACIT-F score was 17.0 (standard deviation (SD) = 7.9) and mean change in FACIT-F score from baseline for placebo was 7.5 (SD = 11.3) [22]. Utilising these estimates, sample size calculations reveal that in a conventional RCT, n = 33 patients per group would be required to detect a mean difference of 8 on the FACIT-F scale between treatment arms with 5% significance (2-sided) and 80% power. With 30% attrition, this would increase to 47 patients per group. Using this same information, except based on the three-cycle aggregated SPT design and assuming there is no period effect, within-patient serial correlation, or treatment × time interaction, the study statistician (PS) designed a computer-based simulation model in SAS statistical software (ver 9.2, SAS Institute Inc., Cary, NC, USA) to estimate the required sample size . Assuming 60% completion of one cycle, 50% two, and 45% completion of all three cycles, then simulations of size N = 10,000 yielded that approximately 21 patients were required at 5% significance and 80% power. To account for likely within-patient serial correlation and potentially worse attrition, the study plans to recruit 40 patients.

### Statistical analysis

There are a number of statistical methods proposed for the analysis of SPTs, with hierarchical Bayesian statistical methods being considered the most appropriate.

The advantages of a Bayesian approach over normal frequentist statistical methods are discussed in detail in Zucker et al. 1997, Schluter and Ware 2005 and Nikles et al. 2011 [10-12]. Briefly, these methods allow: (a) both individual and aggregate analyses to be simultaneously and coherently undertaken–even when the number of completed cycles between patients is variable, (b) they exploit and accommodate natural hierarchies and serial correlations within the study (such as clustering by physician, setting, or location), (c) the outcome variable of interest can take any functional form, (d) confounding variables can easily be introduced, (e) they naturally allow the incorporation of any relevant trial information that may be sourced from elsewhere, and (f) they produce sensible estimates and confidence intervals [10-12]. Most proposed methods for combining simple measures of the difference between active and placebo treatments cannot make such assertions and often assume a normal distribution of differences; an often rich assumption, [12,23] especially with the frequent use of nominal scales [11]. However, testing or the validation of this assumption can be hampered by the small number of cycles a patient may undertake, and so violations are often undetected. Consequences of these assumption violations are imprecise, incoherent and/or impossible estimates; all of which considerably reduce the utility of any pursuant statistical estimate or inference. Bayesian methods are still largely ignored in the medical literature–as there is the perception that such methods are difficult to employ. However, as can be seen in the programme code given by Schluter and Ware, using a freely downloaded computer software package (WinBUGS), this argument holds less ground in modern times.

### Ethical considerations

#### Ethical approval

Written approvals have been obtained from relevant Hospital Research Ethics Committees (Hunter New England HREC, Gold Coast Health Service District HREC, Mater Health Services HREC, Royal Brisbane and Women’s Hospital HREC, St/Vincent’s and Holy Spirit Health HREC) and The University of Queensland Medical Research Ethics Committee prior to study commencement. Informed consent is required for patient trial registration. All patients receive a re-identifiable registration number for the trial CRFs and database.

#### Trial withdrawal and discontinuation of trial medication

Patients can withdraw from the trial at any time without reason or impact on usual care and reason for withdrawal is recorded. The study drug will be discontinued and the participant withdrawn from study if there is a drug-related adverse event or the participant is not well enough to continue the study medicine. If the study drug is discontinued following some unexpected event, it can be restarted at a later date, assuming the participant consents. Only data from completed cycles will be included in the population analysis. The trial will be stopped if DSMB recommend stoppage due to safety concerns.

## Discussion

Cancer is the second most common cause of death in Australia, and the incidence is likely to increase with an ageing population. A prevalent condition associated with cancer and/or its treatment is fatigue with negative consequences on patients and carers quality of life and function.

Evidence for the use of psychostimulants in managing fatigue in cancer is still largely based on non-randomized studies or small studies and in patients with a range of cancer severity (eg [24-26]). In trials, there are mixed results, with one prophylactic placebo-controlled trial of methylphenidate in people undergoing radiotherapy for brain tumors showing no effect in preventing fatigue [27]. Another phase III trial of methylphenidate in patients receiving chemotherapy also found no effect on cancer-related fatigue or QOL, except after a subset analysis for those with more severe fatigue and/or advanced disease [28]. In contrast, Lower et al. in their placebo controlled trial in cancer patients undergoing chemotherapy found a significant improvement in fatigue symptoms [29]. A recent systematic review concluded that preliminary evidence provides partial evidence for the use of psychostimulants to treat cancer-related fatigue [26]. Given the uncertainty around the benefit of psychostimulants in the management of fatigue in patients with advanced cancer, and the high prevalence of this problem and its negative impact on QOL of many patients and their families/carers, it is imperative that further evidence is obtained from well-designed studies [26].

Due to the challenges in recruiting patients with advanced disease into trials, we propose an alternative approach to RCTs while providing high-level evidence on treatment effects. Aggregated SPTs, due to their cross-over design, reduce the required sample size while controlling for confounding.

The main limitation of SPTs in the palliative care population is the possibility of disease progression causing differences between arms of a test cycle. This problem is addressed in the methodology. SPTs are appropriate where (a) the condition remains relatively stable during follow-up period (to the extent that changes observed within replicate pairs cannot be attributed to disease progression) (b) the medication does not alter the pathophysiology of the condition; (c) the medication has a short half-life; and (d) there is validated measurement of effect [9]. In palliative care, single crossover trials have been published [30], but there are few multiple crossover formal SPTs [31]. MPH meets the criteria for utilising an n-of-1 trial method in advanced cancer as (a) the cancer fatigue should remain stable as a short period of follow-up, (b) there is no residual impact on the target symptom once excreted, (c) it has a short half-life and (d) validated measures are used.

SPTs offer two advantages not currently available in palliative care. The first is that the individual patient receives the strongest evidence possible about the effectiveness of the test treatment against the comparator for them. Treatment can be tailored to the individual to allow treatment recommendations. The second is that a series of such individual trials of a given treatment can yield an estimate of the population effect comparable to a RCT, but requiring substantially less numbers of participants to gather that evidence, which in a difficult to recruit population is an important design strength.

This study is the first study to employ aggregated, multiple n-of-1 s in palliative care research, and will not only assess the effectiveness of methylphenidate on fatigue reduction, but will also evaluate the methodological and analytical approaches to conducting SPTs in this population. SPTs require evidence on the proof of concept, and establishment of their place as a practical and reliable research approach in the palliative care population. The availability of this information will influence research policy and change clinical practice by improving availability of high quality evidence in drug studies in difficult to recruit and/or follow patient populations.

## Abbreviations

AKPS: Australian Karnofsky Performance Scale; DSMB: Data and Safety Monitoring Board; FACIT-F: Functional Assessment of Cancer Therapy-fatigue subscale; MPH: Methylphenidate; MMSE: Mini-mental state examination; QOL: Quality of life; RCT: Randomised Controlled Trial; SPT: Single patient (n-of-1) trial.

## Competing interests

The authors have no conflicting interests to declare.

## Authors’ contributions

GM, JH, JN conceived the study. HS was responsible for drafting the manuscript. All authors contributed to the design of the study. All authors contributed and approved the manuscript.

## Authors’ information

Hugh Senior, DPH MSc PhD; Mitchell Geoffrey, MBBS PhD FRACGP FAChPM; Jane Nikles, MBBS PhD; Sue-Ann Carmont, BA (hons); Phillip Schluter, PhD; David Currow, BMed MPH FRACP; Rohan Vora, MBBS FACLPM; Michael Yellard, MBBS PhD FRACGP FAFMM; Meera Agar, MPallCare FRACP FAChPM; Phillip Good, MBBS FRACP FAChPM; Janet Hardy, MD FRACP.

## Pre-publication history

The pre-publication history for this paper can be accessed here:

http://www.biomedcentral.com/1472-684X/12/17/prepub
